# Experimental Study on the Behavior of TiN and Ti_2_O_3_ Inclusions in Contact with CaO‐Al_2_O_3_‐SiO_2_‐MgO Slags

**DOI:** 10.1155/2017/2326750

**Published:** 2017-08-08

**Authors:** S. K. Michelic, C. Bernhard

**Affiliations:** Chair of Ferrous Metallurgy, Montanuniversitaet Leoben, Leoben, Austria

## Abstract

TiN and Ti_2_O_3_ are the predominant inclusion types in Ti-alloyed ferritic chromium stainless steels. In order to ensure the required steel cleanness level, an effective removal of such inclusions in the slag during secondary metallurgy is essential. This inclusion removal predominantly takes place via dissolution of the inclusion in the slag. The dissolution behavior of TiN and Ti_2_O_3_ in CaO-SiO_2_-Al_2_O_3_-MgO slags as well as their agglomeration behavior in the liquid steel is investigated using High Temperature Laser Scanning Confocal Microscopy and Tammann Furnace experiments. Thermodynamic calculations are performed using FactSage 7.0. The behavior of TiN is observed to be completely different to that of oxides. Ti_2_O_3_ dissolves quickly in slags, and its dissolution behavior is comparable to that of other already well examined oxides. In contrast, TiN shows a very intense gas reaction which is attributed to the release of nitrogen during contact with slag. Slags with higher SiO_2_ content show a significantly higher ability for the dissolution of TiN as compared to Al_2_O_3_-rich slags. The gas reaction is found to also significantly influence the final steel cleanness. Despite the easy absorption of TiN in the slag, the formed nitrogen supports the formation of pinholes in the steel.

## 1. Introduction

Titanium-alloyed ferritic stainless steels are a comparable alternative to austenitic stainless steels concerning mechanical properties and corrosion resistance. However, the addition of titanium causes a change in inclusion landscape. Different titanium-containing inclusions form either directly after alloying or during solidification. TiN, Ti_2_O_3_, or Ti(C,N) can precipitate depending on the specific oxygen and nitrogen level. These inclusions interact with other endogenous or exogenous inclusions resulting in inclusion agglomerates or even the formation of clusters, which can finally be the origin of casting problems or surface defects on the final product [1–3].

A previous publication of the authors [4] focused on the detailed metallographic characterization of TiN, TiC, and Ti(C,N) with regard to their size, morphology, and composition. The methods used are manual and automated Scanning Electron Microscopy with Energy Dispersive X-ray Spectroscopy (SEM/EDS) as well as optical microscopy. Additional thermodynamic calculations are performed to explain the precipitation procedure of the analyzed titanium nitrides. The analyses showed that homogeneous nucleation is decisive at an early process stage after the addition of titanium. Heterogeneous nucleation gets crucial with ongoing process time and essentially influences the final inclusion size of titanium nitrides. Tendencies regarding the formation and modification time of titanium-containing inclusions in ferritic chromium steels have been derived, based on different inclusion morphologies, in combination with thermodynamic results.

The present publication continues this research work considering the agglomeration tendency of Ti_2_O_3_ and TiN in the liquid steel as well as their behavior as soon as they get in contact with a slag phase. This situation changes the conditions significantly and leads to different reactions finally also resulting in further changes of the final inclusion landscape as well as the possible appearance of pinholes in the product.

Sharan et al. [5] used Drop Shape Analyses for determining the contact angle between TiN and various ladle slags in the system CaO-Al_2_O_3_-SiO_2_ as well as mold slags. They observed the formation of gas bubbles as soon as the slag got in contact with the TiN. Analyses with a mass spectrometer confirmed that the released gas is nitrogen. Scheller [6, 7] investigated the mass exchange at the interface steel-mold powder. He observed a significant mass exchange, especially dominant for the reaction between SiO_2_ in the slag and Ti in the steel melt. SiO_2_ in the slag is reduced resulting in a decrease of the SiO_2_ content and an increasing Ti_2_O_3_ content in the slag. Furthermore, he confirmed that the Ti-SiO_2_ reaction causes an enrichment of Ti and oxygen in the liquid steel near the steel/slag interface. This is attributed to the formation of a CaO-Al_2_O_3_-SiO_2_-MgO layer.

Based on these previous investigations, the present work focuses on the reactions of TiN and Ti_2_O_3_ in contact with CaO-Al_2_O_3_-SiO_2_-MgO slags and in Ti-stabilized ferritic stainless steel. Thermodynamic calculations are used to estimate the occurring reactions during the steel/slag contact. The inclusion behavior is observed in situ in the High Temperature Laser Scanning Confocal Microscope. Furthermore, remelting experiments in a Tammann Furnace are performed. The differences in reaction behavior of TiN and Ti_2_O_3_ are described and their relevance for the final steel cleanness is discussed.

## 2. Materials and Methods

### 2.1. Thermodynamics

Thermodynamic calculations with FactSage 7.0 using the databases FStel and FToxid are performed to study the reaction of TiN and Ti_2_O_3_ with different slag compositions in the system CaO-Al_2_O_3_-SiO_2_-MgO. Based on available literature [6, 7], particularly the reaction with SiO_2_ is in the focus of interest.  Table 1 summarizes the two slag compositions investigated in the present study. TiN and Ti_2_O_3_, respectively, are continuously added to the slag simulating the interaction between particle and slag.

**Figure 1:**
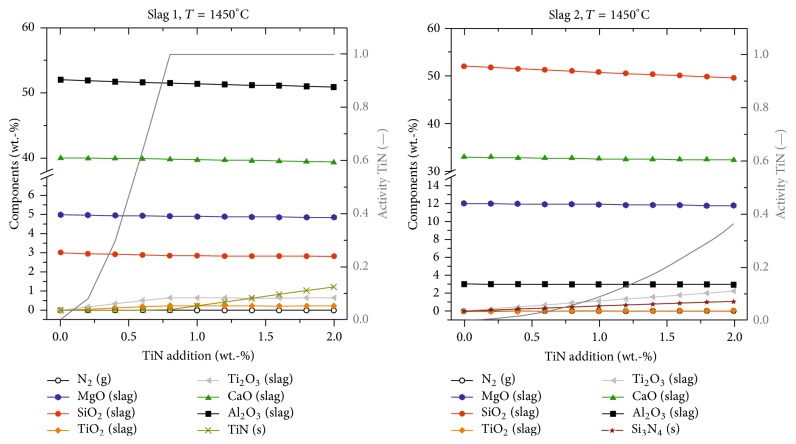
FactSage calculation results for the reaction of TiN in contact with slags 1 and 2.

The calculation results for TiN reacting with slags 1 and 2 at 1450°C assuming equilibrium conditions are summarized in Figure 1. Comparing the behavior of TiN in the two slags, significant differences can be observed:

(i) In slag 1 (Al_2_O_3_-rich slag), the activity of TiN equals 1 after addition of appr. 0.8 wt.-% TiN. As long as the activity is smaller than 1, a reaction between TiN and the slag components takes place. Al_2_O_3_ and SiO_2_ are reduced by 1.15% and 3.4%, respectively. Less than 1 wt.-% of Ti_2_O_3_ and TiO_2_ is formed in the slag. As soon as the activity of TiN equals 1, the TiN content increases continuously with further addition of TiN to a maximum of 1.2 wt.-%. No further reduction of oxidic slag components takes place.
(ii) In slag 2 (SiO_2_-rich slag) the activity of TiN never becomes 1. Thus, a continuous reduction of oxidic slag components, predominantly SiO_2_ (reduced by 5.8%), is observed resulting in the increasing percentage of maximum 2.23 wt.-% Ti_2_O_3_ and 0.04 wt.-% TiO_2_ in the slag with increasing TiN addition up to 2 wt.-%. The prevailing reaction can be described as given in
  (1) $$\begin{array}{c} x\langle \;TiN\;\rangle +\frac{y}{2}\left(\;SiO_{2}\;\right)⟶\left(\;{Ti}_{x}O_{y}\;\right)+\frac{y}{2}\left[\;Si\;\right]+\frac{x}{2}\left\{\;N_{2}\;\right\}. \end{array}$$
(iii) In both cases, nitrogen gas is formed due to the reaction of TiN with slag components.

**Figure 2:**
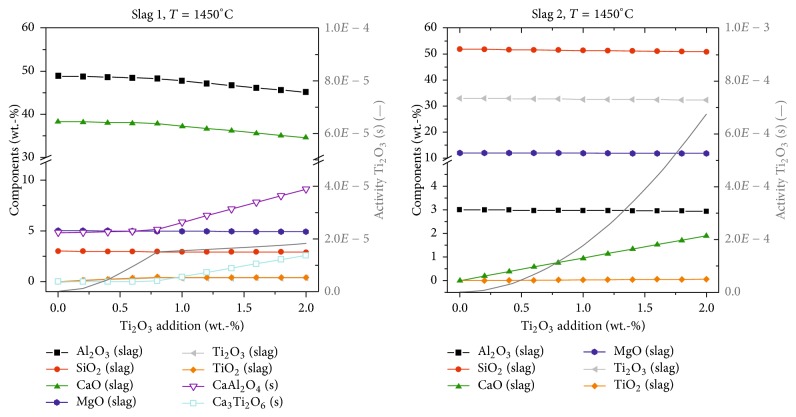
FactSage calculation results for the reaction of Ti_2_O_3_ in contact with slags 1 and 2.

Figure 2 illustrates the behavior if Ti_2_O_3_ gets in contact with slags 1 and 2. The main results can be summarized as follows:

- A Ti_2_O_3_ activity of 1 is reached in neither slag 1 nor slag 2. The reaction with slag components starts immediately.
- The behavior of Ti_2_O_3_ in slag 1 shows that up to an addition of app. 0.8% wt.-Ti_2_O_3_ oxide slag components get slightly reduced (Al_2_O_3_ by 1.17%, SiO_2_ by 2.24%, and CaO by 1.13%) and Ti_2_O_3_ and TiO_2_ are formed with a total amount of 0.76 wt.-% after adding 2 wt.-% Ti_2_O_3_ in the slag. For higher Ti_2_O_3_ additions, the precipitation of CaAl_2_O_4_ and Ca_3_Ti_2_O_6_ starts due to a further reduction of slag components Al_2_O_3_ and CaO up to a total amount of 11.7 wt.-% of precipitated CaAl_2_O_4_ and Ca_3_Ti_2_O_6_.
- For slag 2 practically no reduction of oxide slag components is found except a slight reduction of SiO_2_ by 2.24%. Ti_2_O_3_ almost goes directly to the slag.
- In contrast to the reaction of TiN with slags 1 and 2, no gas formation is predicted, neither in slag 1 nor in slag 2.

It can be concluded that slags with higher SiO_2_ content show a higher ability for the dissolution of TiN compared to Al_2_O_3_-rich slags. A variation in the Al_2_O_3_ content only showed a minor influence.

### 2.2. Experimental Methods

Based on the thermodynamic calculations, two different experimental methods are applied in order to study the behavior of TiN and Ti_2_O_3_ in contact with different slags as well as the agglomeration tendency of these inclusion types in the Ti-stabilized ferritic stainless steel 1.4520.

#### 2.2.1. High Temperature Laser Scanning Confocal Microscopy

High Temperature Laser Scanning Confocal Microscopy (HT-LSCM) enables the in situ observation of inclusion reactions at steelmaking temperatures. The principal set-up consists of a Confocal Scanning Laser Microscope type VL2000DX from Lasertec and a high temperature furnace type SVF17-SP from the manufacturer Yonekura. A laser with a wavelength of 408 nm is used as a light source. Since this wavelength is below the thermal radiation spectrum of the observed sample, the image contrast is of high quality. Heating rates up to 1200°C/min and cooling rates of 1000°C/min are possible. More details on the HT-LSCM, its background, and application are given in [8, 9].

**Figure 3:**
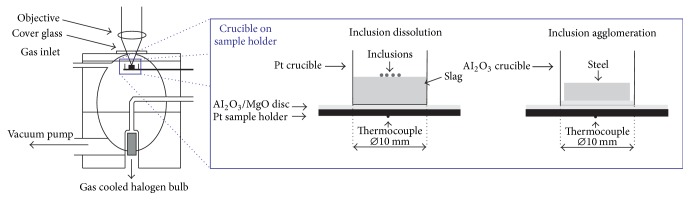
Schematic illustration of the high temperature chamber as well as the experimental set-up for slag experiments.

Figure 3 demonstrates the main experimental set-up of the high temperature chamber. The attached high temperature furnace shows an elliptic, gold-coated inner contour. The halogen lamp is situated in the bottom focal point. The sample holder with the crucible containing the sample is located in the top focal point of the ellipse. The temperature is measured with a thermocouple fixed at the bottom side of the sample holder. The position of the thermocouple inevitably results in a temperature difference between the sample surface and the thermocouple. Therefore, an accurate temperature calibration is necessary before the experiment. All experiments are carried out under inert argon atmosphere.

Two different kinds of experiments are performed in the present study:

- *Inclusion agglomeration in the liquid steel:* the steel sample is placed in an Al_2_O_3_ crucible and heated up to a temperature of 1500°C using a heating rate of 1000 K/min and then heated up to the experimental temperature of 1600°C with a heating rate of 10 K/min. An alumina disc is placed under the steel disc in order to avoid the attachment of the steel disc onto the crucible. When the liquidus temperature of the steel is reached, inclusions are starting to emerge from the bulk to the steel surface and their agglomeration behavior can be studied. Only a small liquid pool is formed based on the principle of “concentric solidification” [10]. To avoid the formation of a complete steel droplet due to the interfacial tension between refractory and liquid steel it is important to keep the outer ring of the sample solid.
- *Inclusion dissolution in a slag phase:* inclusion particles are placed on a premelted synthetic slag in a platinum crucible. The slag is mixed out of the raw powders and first premelted in an electric furnace. After crushing, the chemical composition is determined using XRF analyses. The slag is then remelted a second time in the HT-LSCM before the particles are placed on the top. The slag with the particles is then heated up to the experimental temperature of 1450°C using a heating rate of 1000 K/min up to 1400°C followed by 10 K/min up to 1450°C in order to avoid exceeding the temperature. The high initial heating rate is needed to ensure that inclusion dissolution hardly starts before the experimental temperature is reached.

Many studies on the dissolution of common oxides like Al_2_O_3_, MgO, and MgOAl_2_O_3_ with the LSCM technique are available in literature [11–13]. In most of these studies, the governing dissolution mechanism has also been examined next to absolute dissolution time. For this purpose only one single, round shaped particle with a diameter of appr. 400 *μ*m is placed on the slag in order to determine the diameter reduction with time. With such a set-up it is also possible to evaluate normalized dissolution curves (whether they show an S-shaped or parabolic tendency) and diffusion coefficients can be calculated.

However, the focus of the present study is not to determine the responsible dissolution mechanism but to display difference in reaction behavior of TiN and Ti_2_O_3_ in contact with the slags. Therefore, particle sizes comparable to inclusion sizes detected in steel samples are used for the investigations. Additionally, not only is one particle used, but several inclusions are employed. To the best knowledge of the authors, results of in situ studies on the TiN and Ti_2_O_3_ dissolution in CaO-Al_2_O_3_-MgO-SiO_2_ have not been published so far. Based on the described set-up it is only possible to determine a qualitative comparison; absolute dissolution times cannot be determined.

#### 2.2.2. Tammann Furnace Experiments

The Tammann type furnace (Ruhrstrat HRTK 32 Sond.) is a high temperature electric resistance furnace that can be heated to 1700°C. Due to the carbon heating tubes inside the furnace and their reaction with the residual oxygen, the final oxygen content in the furnace vessel is extremely low (0.001 ppm). All experiments are conducted under an inert gas atmosphere. Further details to the experiment itself and other possible applications can be found in [14, 15].

**Figure 4:**
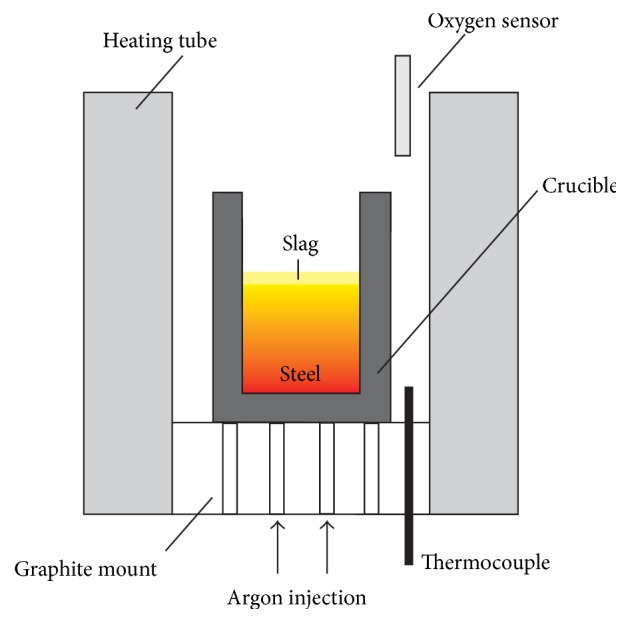
Schematic illustration of the Tammann Furnace used for laboratory experiments.

For the experiments approximately 260 g of steel (composition given in Table 2) is melted in a MgO crucible. On top of steel approximately 40 g of slag are placed. The ratio between steel and slag in the experiment is based in industrial practice applied for the investigations in a previous publication [4]. The slags used are the same as for the LSCM experiments (see Table 1). However, it has to be indicated that due to the use of a MgO crucible slag composition could lead to a systematic influence in all experiments because of slag-refractory interactions. The whole experimental set-up is schematically shown in Figure 4. Steel and slag are heated up to a temperature of 1600°C with a subsequent holding time of 30 min. After rapid cooling of the sample, the reactions and modifications of inclusions in the steel, with the refractory material as well as at the steel/slag interface, are studied. For this purpose, the whole sample and the crucible are cut along the axis in order to be also able to study the steel/refractory interface. The other half of the steel sample is prepared metallographically to investigate the inclusion landscape after the experiment. Through a comparison with the original inclusion landscape, determined using automated SEM/EDS analyses [4], conclusions regarding inclusion modification caused by steel/slag interaction can be drawn.

**Figure 5:**
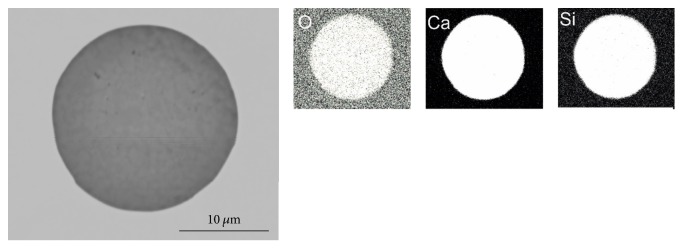
Typical CaO-SiO_2_ inclusion in steel 1.4520 at an early stage of the production process.

**Figure 6:**
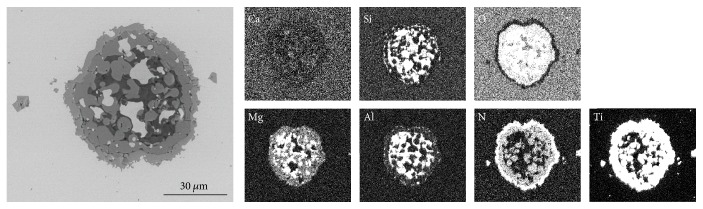
Complex oxide inclusion surrounded by a TiN layer in steel 1.4520.

Before remelting the steel samples in the Tammann Furnace, the inclusion landscape can be characterized as follows:

- The predominant inclusion type in the investigated steel 1.4520 is TiN. Several examples of different TiN morphologies are given in a previous publication from the authors [4]. Pure Ti_2_O_3_ is hardly found.
- Some complex oxide inclusions are also detected. Examples for such oxides are given in Figures 5 and 6. These findings agree well with the results published by Park [16] who analyzed an austenitic stainless steel and also observed CaO-SiO_2_ inclusions with parts of MgO and Al_2_O_3_ at an early stage of the production process. In the present case, pure CaO-SiO_2_ inclusions are modified with ongoing processing with Mg and Al and finally a TiN layer is formed around the oxide inclusions (see Figure 6).

## 3. Results and Discussion

### 3.1. Inclusion Agglomeration in the Steel

**Figure 7:**
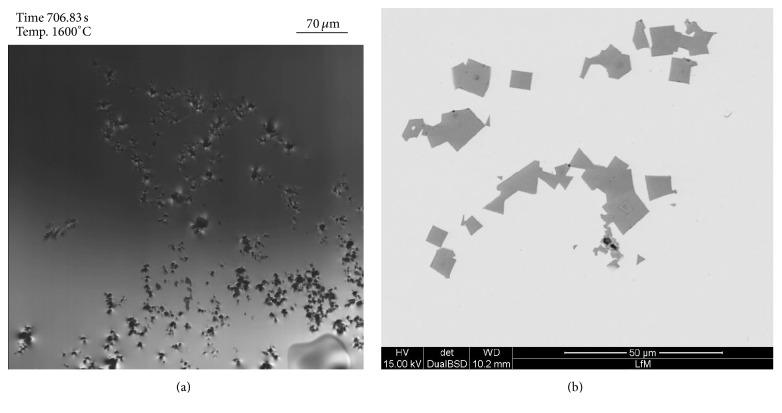
TiN agglomeration in the HT-LSCM at 1600°C (a) and TiN agglomerates in the solidified sample (b).

Figure 7 illustrates the agglomeration behavior of TiN in the liquid steel.  Figure 7(a) shows an in situ image of the HT-LSCM. The SEM image at Figure 7(b) demonstrates agglomerated inclusions in the resolidified sample. Originating from an inclusion size smaller than 10 *μ*m ECD for single TiN particles, the agglomeration causes a significant increase in the inclusion size resulting in agglomerates larger than 50 *μ*m in width. The original inclusion landscape in these steel samples has already been analyzed in detail in a previous publication of the authors [4]. The predominant inclusion type (more than 90% of the overall count) is TiN. Since the chemical composition of inclusions cannot be analyzed during the LSCM experiment, the only possibility of distinguishing between different inclusion types is based on the experience of previous EDS investigations. Additionally often reported differences in the morphology between nitrides and oxides can also be used as an indication for determining the observed inclusion type. Both approaches are used in the present study.

**Figure 8:**
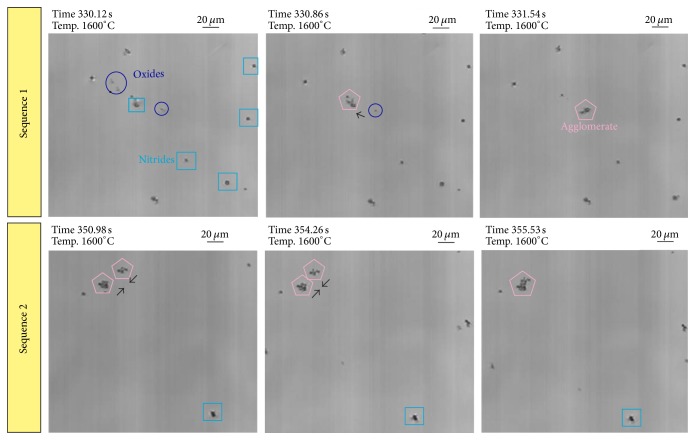
HT-LSCM sequences of inclusion agglomeration processes at 1600°C in the steel 1.4520.

**Figure 9:**
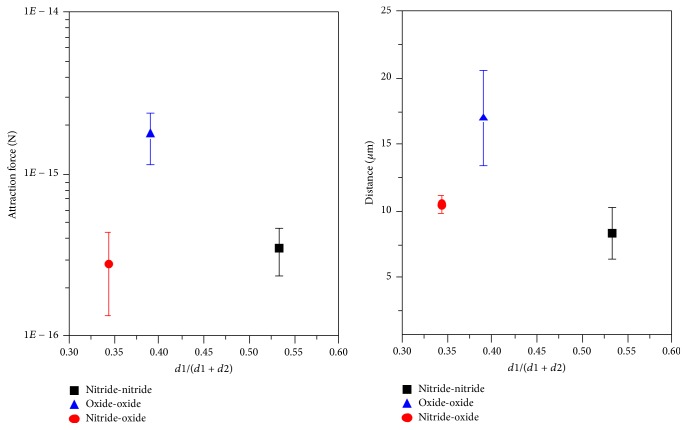
Comparison of attraction forces and distance between three different particle combinations.

Two agglomeration processes of oxides and nitrides at 1600°C are demonstrated in Figure 8. Inclusion types are distinguished by visual comparison with previous SEM/EDS investigations of this steel grade, which showed that TiN is mostly characterized by a very rectangular shape, whereas oxides mostly appear in globular form. All observed inclusions are solid in this case. As shown in sequence 1, the agglomeration of 4 inclusions, 3 oxides and 1 nitride, happens in less than one second. The velocity of inclusions increases with decreasing distance between the particles and an increasing attraction force is observed before the final agglomeration happens. The formed agglomerate then acts as an attractor for further particles. Sequence 2 illustrates the formation of an agglomerate with an approximate size of 30 *μ*m in diameter. The agglomerate consists of 10 to 20 small nitrides and oxides. Based on the LSCM experiments, attraction forces between different particle combinations are calculated applying the procedure described in two publications by the authors [16, 17].  Figure 9 shows a comparison of calculated attraction forces between three different particle combinations (oxide-oxide, oxide-nitride, and nitride-nitride). Since the inclusion size significantly influences attraction, only interactions with similar *d*1/(*d*1 + *d*2) ratio have been analyzed in the calculations. The used density values are 4490 kg/m^3^ for Ti_2_O_3_ (globular shaped) and 5900 kg/m^3^ for TiN (disc shaped). The results indicate a stronger attraction force for oxide-oxide combination compared to oxide-nitride and nitride-nitride. Additionally, the attraction distance between two oxides is stronger than between oxide-nitride and nitride-nitride.

The oxides observed in Figure 8 are very small compared to those shown in Figures 5 and 6. Since the observed sample size in the HT-LSCM is very small, it is rather unlikely to find single inclusions with sizes >30 *μ*m ECD in these steel samples. However, the SEM image in Figure 7 visualizes small black areas between or at the borders of TiN. These are small oxides which can also promote the heterogeneous nucleation of TiN in this steel. Details on this nucleation can be found in the authors' previous publication [4].

### 3.2. Reduction of TiN in Contact with a Slag Phase

**Figure 10:**
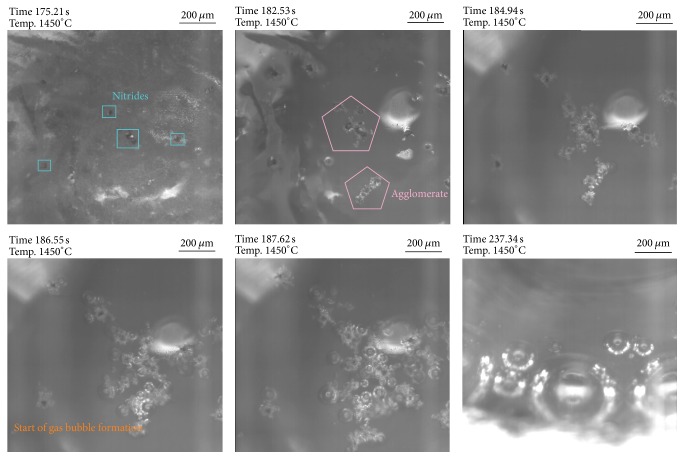
HT-LSCM sequences of the reaction behavior of TiN in contact with slag 1.

**Figure 11:**
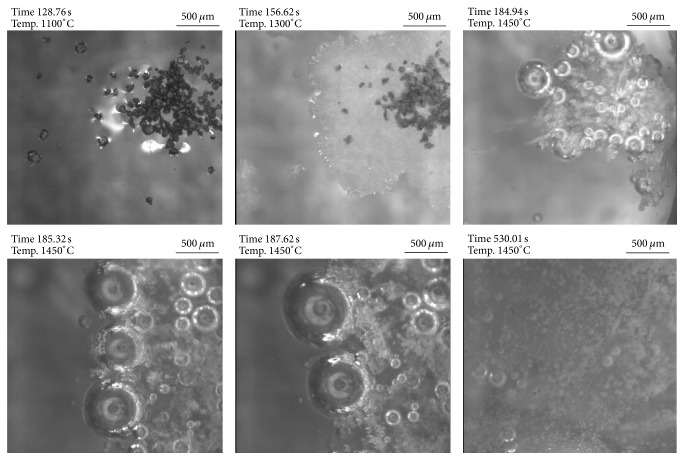
HT-LSCM sequences of the reaction behavior of TiN in contact with slag 2.

Thermodynamics revealed that a reaction of TiN with slag components takes place in both investigated slags forming nitrogen gas. However, due to the higher Si-content in slag 2, the TiN reduction should be much more effective. Figures 10 and 11 summarize the TiN behavior in slags 1 and 2 in the HT-LSCM at a temperature of 1450°C. For slag 1 (see Figure 10) TiN is clearly visible at the slag surface at the beginning tending to agglomerate within less seconds. Already after approximately 10 seconds the formation of gas bubbles is clearly visible. Bubble formation intensifies with ongoing time but slows down again after 50 seconds and totally stops after around 60 seconds. Due to the reduction of TiN causing an increase of Ti_2_O_3_ in the slag, the visibility worsens with ongoing experimental time.

In the case of contact between TiN and slag 2 (see Figure 11) at experimental temperature, TiN reduction also starts immediately. In contrast to slag 1 the observed gas formation is much more intense and also holds on for a much longer time, due to the high Si-content in the slag. After 400 seconds of experimental time, still a significant amount of gas bubbles can be observed. The experiment is stopped at this point. This observation of TiN behavior in the HT-LSCM is in very good agreement with the results of thermodynamic calculations. However, of course also kinetic aspects at the slag-inclusion interface can essentially influence the dissolution behavior and especially the governing dissolution mechanism. The latter was not studied in detail in the present study as the standard experiment using only one large particle in the slag experiment would be needed for this purpose.

### 3.3. Dissolution of Ti_2_O_3_ in Contact with a Slag Phase

**Figure 12:**
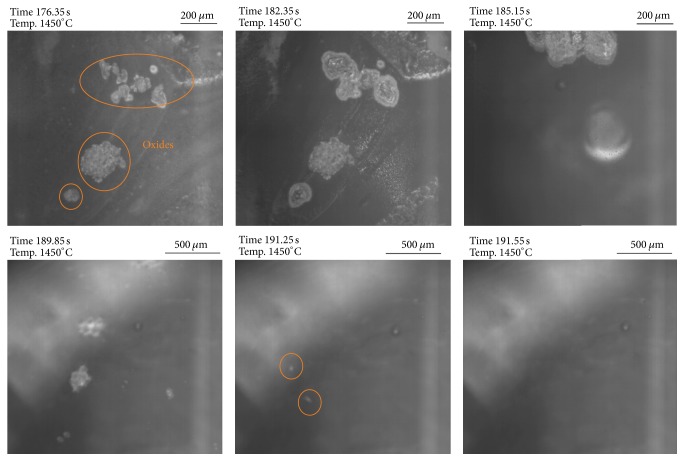
HT-LSCM sequences of the dissolution behavior of Ti_2_O_3_ in contact with slag 1.

**Figure 13:**
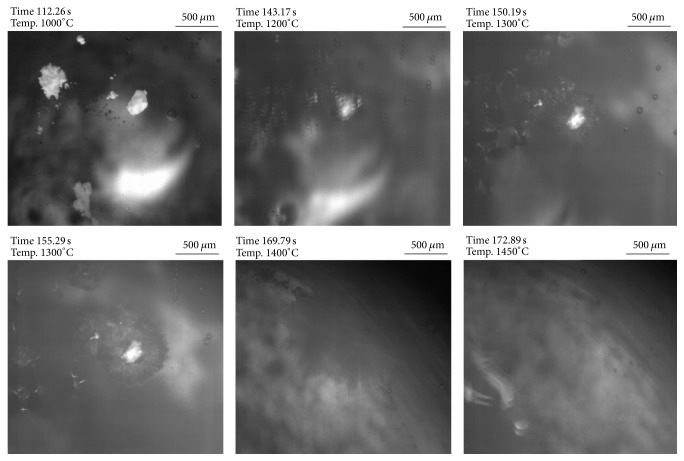
HT-LSCM sequences of the reaction behavior of Ti_2_O_3_ in contact with slag 2.

According to thermodynamics the behavior of Ti_2_O_3_ should be completely different than that of TiN in the investigated slags.  Figure 12 demonstrates the dissolution behavior of Ti_2_O_3_ in contact with slag 1. At the experimental temperature, all added Ti_2_O_3_ is fully dissolved after approximately 15 seconds. The dissolution of Ti_2_O_3_ particles in slag 2 is shown in Figure 13. In this case, Ti_2_O_3_ dissolution already starts before the experimental temperature is reached. At 1450°C all Ti_2_O_3_ is already dissolved in the slag phase. One reason for the early start of the Ti_2_O_3_ dissolution can be that there is no reaction with other slag components as predicted by thermodynamics. In both cases in contrast to the behavior of TiN in the two slags no gas bubble formation is observed which also confirms the calculation results. In total, the reaction time of Ti_2_O_3_ with the slags is significantly shorter compared to the TiN reaction.

All observed reactions in the HT-LSCM are briefly summarized in Table 3.

### 3.4. Formation of Pinholes

**Figure 14:**
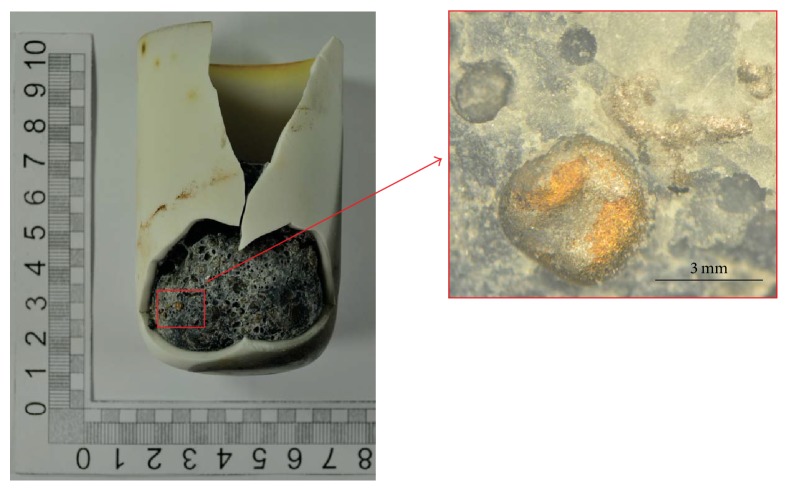
Crucible after the remelting experiment in the Tammann Furnace exemplified for an experiment with slag 1.

**Figure 15:**
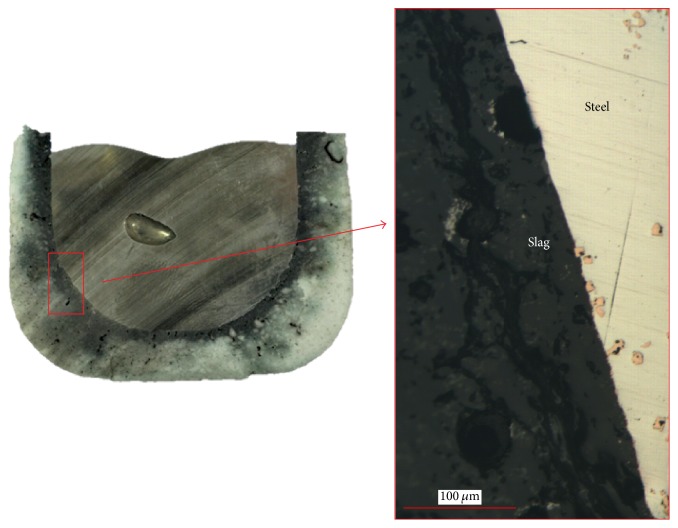
Cross section of the sample in the crucible after the Tammann Furnace experiment illustrating the attachment of TiN at the steel/slag interface.

**Figure 16:**
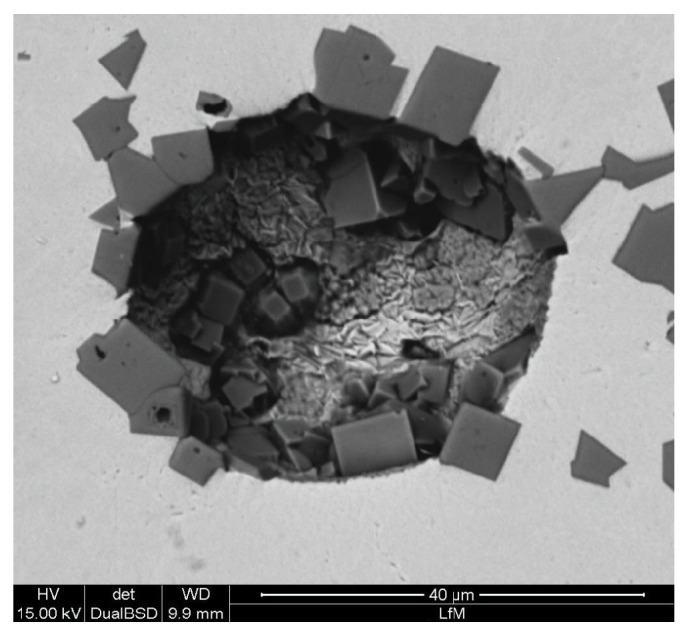
Pinhole with attaching TiN in the steel sample after remelting in the Tammann Furnace with slag 2.

Owing to the presence of TiN in combination with oxides in the liquid steel and the occurring reactions if an inclusion gets in contact with a slag phase, another defect can emerge in the steel matrix.  Figure 14 shows a broken crucible after the Tammann Furnace experiment. At the steel surface several golden shining blisters can be found. A cross section of the remelted sample is displayed in Figure 15. A change in color of the crucible material near the steel sample is clearly visible, indicating the infiltration of slag in the refractory material. A closer examination of the interface confirms that, in both cases (remelting with slags 1 and 2), a thin slag layer is present next to steel covering the whole interface. Additionally, several TiN inclusions are observed at the steel-slag interface. These findings also confirm the described reactions at the steel-slag interface as well as the release of nitrogen through the reduction of TiN as soon as the inclusion gets in contact with the slag. A typical defect resulting from these reactions is illustrated in Figure 16. Sample pinholes appear inside the molten steel which can act as potential nuclei for the attachment of TiN. Such a defect could not be found in the steel before remelting in the Tammann Furnace. Thus, its appearance can be directly attributed to the steel-slag reactions which is also in good accordance to thermodynamics as well as dissolution experiments. No significant difference in the appearance or number of pinholes between samples remelted with slag 1 or slag 2 is observed.

**Figure 17:**
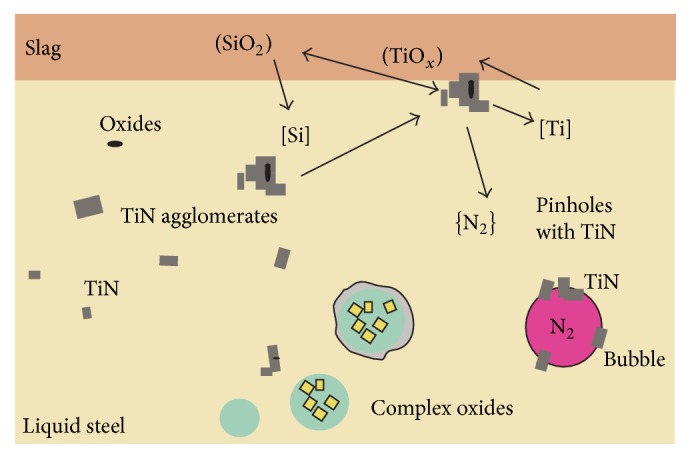
Schematic illustration of occurring reactions if TiN gets in contact with the slag phase.

Figure 17 summarizes the detected inclusion landscape in the investigated steel 1.4520 and the possible changes caused by steel-slag interactions. The proceeding reactions can be divided into the following steps:

1. TiN forms in the steel due to homogeneous or heterogeneous nucleation. The latter is the predominant inclusion type in this steel grade. The presence of small single TiO*x* only plays a minor role. Both types are solid inclusions which generally have a strong tendency for agglomeration.
2. Agglomerates of TiN approach the steel-slag interface and react with slag components, predominantly with SiO_2_. The Ti_2_O_3_ content in the slag increases.
3. TiN are reduced resulting in the formation of nitrogen gas bubbles which subsequently form pinholes in the solidifying steel.
4. Formed pinholes tend to act as potential nuclei for the attachment of TiN.

## 4. Conclusion

The present study focused on the behavior of TiN and Ti_2_O_3_ inclusions in the steel 1.4520 in contact with two different slag compositions. Thermodynamic calculations and different experimental techniques are used to study the reactions and interactions at the steel-slag interface. The following conclusions are drawn:

- Thermodynamic calculations predict a totally different behavior of TiN and Ti_2_O_3_ in contact with the investigated slags: slag 1 rich in Al_2_O_3_ and a SiO_2_-rich slag 2.
  - TiN gets stable in slag 1 with increasing TiN addition stopping the further reduction of oxidic slag components. In slag 2, a continuous reduction of oxidic slag components is observed, primarily due to the presence of SiO_2_. Nitrogen gas is formed due to the reaction of TiN with slag components in both cases.
  - In contrast, for the reaction with Ti_2_O_3_ with slags 1 and 2, no gas formation is predicted.
- By using the HT-LSCM, TiN and Ti_2_O_3_ agglomeration in the liquid steel as well as the reduction or dissolution of TiN and Ti_2_O_3_ in the slag phase could be studied in situ:
  - Attraction forces have been calculated for different particle combinations. The attraction force between two oxides proved to be higher than nitride-nitride or nitride-oxide combination. Since TiN represents the majority of inclusions in the observed steel grade, also TiN agglomerations were predominant in the observations.
  - The reduction of TiN in contact with slags 1 and 2 resulting in the formation of nitrogen gas is confirmed by the experiments. TiN reduction is observed to take significantly longer compared to the dissolution of Ti_2_O_3_ in the investigated slags.
- The release of nitrogen due to the occurring reactions can also promote the formation of pinholes which subsequently can act as source for the attachment of TiN. This has been verified by experiments in the Tammann Furnace remelting steel samples with slags 1 and 2.
- Slags with higher SiO_2_ content show a significantly higher ability for the dissolution of TiN compared to Al_2_O_3_-rich slags. However, a compromise between effective dissolution and other involved reactions has to be found, since intense TiN dissolution also can promote the formation of pinholes in the steel.

## Figures and Tables

**Table 1:** Investigated slag compositions.

	CaO [wt.-%]	SiO_2_ [wt.-%]	Al_2_O_3_ [wt.-%]	MgO [wt.-%]
Slag 1	40.0	3.0	52.0	5.0
Slag 2	33.0	52.0	3.0	12.0

**Table 2:** Composition of the investigated steel grade 1.4520 [4].

	C wt.-%	Cr wt.-%	Ti wt.-%	N wt.-%	Mn wt.-%
Min.	—	16.0	0.3	—	—
Max.	0.025	18.0	0.6	0.015	0.5

**Table 3:** Summary of observations in the HT-LSCM.

		TiN	Ti_2_O_3_
Agglomeration		↑↑	↑↑↑

Gas bubble formation		Yes	No

Reaction		Reduction	Dissolution Precipitation of CaAl_2_O_4_ and Ca_3_Ti_2_O_6_

Reaction rate	Slag 1	↑↑	↑↑
	Slag 2	↑↑↑	↑↑↑

Reaction time		≥1 min	<1 min
